# Quality specification of ice creams produced with different homofermentative lactic acid bacteria

**DOI:** 10.1002/fsn3.3762

**Published:** 2023-10-12

**Authors:** Gökhan Akarca, Mehmet Kilinç, Ayşe Janseli Denizkara

**Affiliations:** ^1^ Department of Food Engineering, Faculty of Engineering Afyon Kocatepe University Afyonkarahisar Turkey

**Keywords:** fatty acids, fermentation, functional properties, ice cream, *Lactobacillus* spp.

## Abstract

This study investigated the changes in the physicochemical, microbiological, textural, and nutritional values of ice cream produced by various methods with the addition of different lactic acid bacteria. Adding lactic acid bacteria to the ice cream mix caused a decrease in firmness, consistency, cohesiveness, index of viscosity, pH, a_w_, first drop, complete melting, and overrun values (*p* < .05). These decreases were more pronounced in the samples to which lactic acid bacteria were added before mix maturation (*p* < .05). Firmness and consistency values varied between 15.11–16.26 (g) and 374.58–404.91 (g s), respectively, in the samples to which lactic acid bacteria were added before maturation. No significant effect of the addition of lactic acid bacteria to the ice cream mix on the L*, a*, and b* values of the bacteria before or after mix maturation was detected (*p* > .05). The L* values of the samples varied between 88.91 and 83.36, a* values between 0.76 and 1.32, and b* values between 6.57 and 8.38. An increase was detected in the amount of organic acid (excluding formic acid) in the samples produced with the addition of different lactic acid bacteria (*p* < .05). The number of fatty acids in the samples varied depending on the lactic acid addition and the production method; the rate of this change was generally higher in the samples with added lactic acid bacteria after mix maturation (*p* < .05). In particular, the amounts of short‐ and medium‐chain fatty acids increased in the samples with lactic acid bacteria added after mix ripening, compared to the control sample.

## Graphical abstract

Ice cream, not typically considered a fermented dairy product, can be converted into one thanks to the lactic acid bacteria added to it, improving and altering its textural, sensory, and microbiological properties. The addition of lactic acid bacteria can also raise ice cream's functional and nutritional values. This study highlighted and examined the characteristics of ice cream made in various methods by adding various lactic acid bacteria.

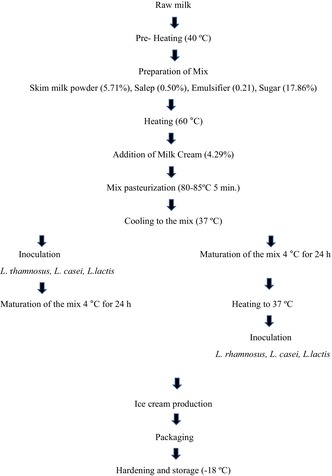

## 1 INTRODUCTION

Due to its distinctive flavor qualities, cooling impact, and high nutritional content, people of all ages consume ice cream, a significant dairy product, all throughout the world (Durmaz et al., 2020; Goktas et al., 2022). The main ingredients in an ice cream mixture include milk, stabilizers, emulsifiers, flavorings, vegetable oils, and fruit. Moreover, ice cream occasionally contains ingredients including probiotic bacteria, pulp, dietary fibred, food colors, and artificial sweeteners (Iahtisham‐Ul‐Hag et al., 2023; Marshall et al., 2003; Senanayake et al., 2013).

Ice cream is a food product with extremely rich nutritional value. The nutritional value of ice cream directly depends on the nutritional values of the ingredients that make up the ice cream. Ice cream also contains all the nutrients that milk has. In addition, ice cream contains 3–4 times more fat and 12%–16% more protein than milk. In addition to these, it is a product richer than milk due to the addition of additives such as fruit, nuts, and eggs (Arbuckle, 2011; Mohammed et al., 2020).

In recent years, consumer perception of nutritious, functional, and natural foods has led ice cream manufacturers to develop newer and healthier products (Batista et al., 2019; Cruz et al., 2009).

Functional foods are those whose ingredients improve the health of the consumers. The demand for functional foods and additives has dramatically expanded globally during the past 15–20 years (Goktas et al., 2022; Halsted, 2003). Today, functional properties can be improved with additives added to many foods product. Milk and foods produced using milk are the most used foods in terms of containing functional properties. One of these products is ice cream; it has excellent potential as a functional food because it allows adding additives with many functional properties (Ozturk et al., 2018).

Fermented products are the first processed foods in human history. Many products, such as milk, meat, and fruit/vegetables, are still widely produced and consumed (Sozeri Atik et al., 2021; Yilmaz et al., 2022). Fermented milk products, which have an important place among fermented products, are highly nutritious, traditional, and/or universal foods consumed with pleasure around the world (Petrova et al., 2021). Depending on the type of milk used during their manufacturing, they are categorized into variants; moreover, the physicochemical, microbiological, and/or production technologies used to produce them cheeses, fermented beverages, yogurt, kefir, ayran, sour cream, and acidofil milk can be counted among the main products made from fermented milk (Shiby & Mishra, 2013).

Today, apart from the products obtained by freezing yoghurts obtained with the addition of different bacteria, there is no real fermented ice cream production. These products are in demand by the consumer due to the presence of probiotic bacteria and aroma difference they contain (Alamprese et al., 2002; Davidson et al., 2000).

In addition, in recent years, ice creams produced by using soy or sesame milk instead of cow's milk enriched with probiotic bacteria are also finding buyers in the market (Ghaderi et al., 2021). There are also studies on the adaptation of compounds obtained from some plants and their roots to the ice cream industry by using different processing methods (Shadordizadeh et al., 2023; Shahi et al., 2021). However, today, there is no real ice cream produced using dairy animal's milk and fermented with lactic acid bacteria.

Ice cream, which is not typically thought of as a fermented dairy product, can be converted into one thanks to the lactic acid bacteria added to it, improving and altering its textural, sensory, and microbiological properties. The addition of lactic acid bacteria can also raise ice cream's functional and nutritional values. The characteristics of ice cream made in various methods by adding various lactic acid bacteria were highlighted and examined in this study.

## 2 MATERIALS AND METHODS

### 2.1 Materials

The study's ingredients, including the cream, salep, granulated sugar (beet sugar), emulsifier (E 471), and cow's milk, were purchased at a local shop in the province of Afyonkarahisar.

### 2.2 Lactic acid bacteria

In this research; *Lactobacillus casei* (ATCC 393), *Lactobacillus rhamnosus* (ATCC 53103), and *Lactobacillus delbrueckii* spp. *lactis* (ATCC 12315) strains were used.

### 2.3 Preparation of lactic acid bacteria strains

MRS broth (Merck, 110,661, Germany) was used to inoculate the lactic acid bacteria (*L*. *casei*, *L*. *rhamnosus*, and *L*. *lactis*) that were employed in the production process before they were cultured at 37°C for 48 h. All strains had a 10‐min incubation at 22°C following the incubation time. The mix was centrifuged at 16424 *g*, and the residue (pellet) at the bottom was separated (Goktas et al., 2022).

### 2.4 Ice cream production

**FIGURE 1:**
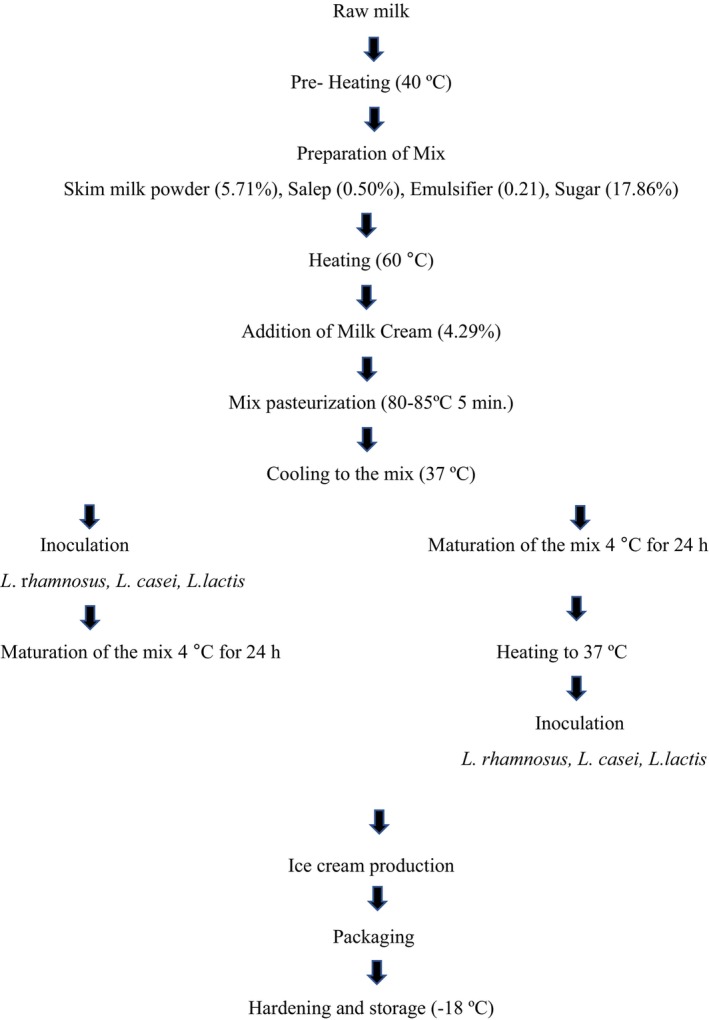
Ice cream production.

Procedures used to make ice cream in the study (Figure 1) were modified from those Kilinc et al. (2022) and Goktas et al. (2022) had previously employed. Two different production methods were carried out: in the first one, adding lactic acid bacteria to the mixture the mixture was then matured. In the second, lactic acid bacteria were added to the mix after maturation. In this way, a total of seven different samples were produced. Subsequently, the prepared mixtures were processed into ice cream in an ice cream machine (CRM‐GEL 25C, Italy) at −5°C. Exactly 250 *g* of each mixture was placed into sterile glass containers and hardened at −24°C. The acquired samples were kept at −18°C until the analysis was finished (Kilinc et al., 2022).

### 2.5 Ice cream mix texture

Ice cream mix firmness, consistency, cohesiveness, and viscosity index values were tested using back extrusion equipment and a TA.XT Plus Texture Analyzer (Stable Micro Systems, Godalming, Surrey, UK) (Sert et al., 2017).

### 2.6 pH value

A homogenizer was used to combine ice cream samples with 1/10 sterilized distilled water (Daihan Wisestir, HS‐30 T, South Korea). A pH meter was then used to measure the pH values (Hanna, HI 2215 pH/ORP).

### 2.7 Water activity (a_w_)

Using a water activity analyzer, the water activity values of the ice cream samples were determined (Novasina LabTouch‐aw, Lachen, Switzerland) (AOAC, 2016).

### 2.8 Color values (L*, a*, b*)

According to the hunter color measurement system, the color values of the samples were assessed using a colorimeter (Minolta Co.) (Akalın et al., 2008).

### 2.9 Lactic acid bacteria count

Lactic acid bacteria counts added to ice cream mixes were performed on man rogosa sharpe agar (110,660, Merck, Germany) (MRS‐Cys‐BPB) (Ngamsomchat et al., 2022) altered with 50 mg of L. casei/L Vancomycin and 50 mg of cysteine bromophenol blue for *L*. *plantarum* (LabM, UK) was added to man rogosa sharpe agar (MRS‐VanC) (Hatakka et al., 2008), *L*. *delbrueckii* spp. For lactis, Man Rogosa Sharpe agar (110,660, Merck, Germany) was used (Shori et al., 2022). Before analysis, serial dilutions were prepared from the samples using 0.1% buffered peptone water (107,228, Merck, Germany) using spread plate methods Anaerocult A (113,829, Merck, Germany) was added to planted petri dishes in anaerobic conditions, jars (116,387, Merck, Germany) were incubated in an oven at 37°C for 72 h (Mena & Aryana, 2012).

### 2.10 First dripping time

The wire strainer on the tared glass containers was used to catch a sample of ice cream (10 *g*), which was then allowed to melt at 20°C. As the ice cream began to melt, the first drops began to fall, the stopwatch was turned on (Shahi et al., 2021).

### 2.11 Complete melting time

A 500 mL glass beaker containing hard ice cream samples from the freezer (−18°C) was placed to thaw at 20°C for the time required for the ice cream to completely thaw. The exact time (minutes) the melting was complete was recorded (Guven & Karaca, 2002).

### 2.12 Overrun

Ice cream samples were poured into a tared glass measuring tape of appropriate size. A beaker was used to defrost the same sample amount of ice cream that had been put in the other container. A clean measuring cylinder was used to transfer the molten mixture to the same volume, and it was then weighed once more (Jimenez‐Florez et al., 1993).

### 2.13 Organic acid

An HPLC was used to calculate the organic acid levels of the ice cream samples (Shimadzu Prominence). Subsequently, 4‐g samples were taken from the samples, 20 mL of 0.01 N H2SO4 was added. Following a 0.45‐μm filter and vortexing, it was introduced into the system (Guzel Seydim et al., 2000). System features used were the following: CBM: 20ACBM; Detector: DAD (SPD‐M20A); Column Furnace: CTO‐10ASVp; Pump: LC20 AT; Autosampler: SIL 20ACHT; Computer Program: LC Solution; Column: ODS 4 (250 mm*4.6 mm, 5 μm) (GP Sciences, Inertsil ODS‐4, Japan); and Mobile phase: orthophosphoric acid was used to bring the pH of ultrapure water to 3 (Aktas et al., 2005).

### 2.14 Free fatty acids

A GC/MS was used to analyze the fatty acids of the samples (AGILENT 5975 C AGILENT 7890A GC). An MSDCHEM program and DB WAX (50*0.20 mm, 0.20 μm) column were used in the device. The initial temperature of the furnace was determined as 80°C; after a 4‐min waiting period, at a rate of 13°C per minute, it was raised to 175°C. It was kept at this temperature for 27 min. Later, 215°C was reached with an increase of 4°C per minute. It was held there for 5 min. After that with an increase of 4°C each minute, 240°C was reached, and this temperature was maintained for 15 min. Detector and injector temperature was 240°C. The injector and detector temperatures were set to 240°C and the injection volume to 1 L. HCl at a concentration of 1.5 M was used as the derivatizer in the analyses, the derivatization temperature was 80°C, and the derivatization time was 2 h (Bardakcı & Secilmis, 2006).

## 3 RESULTS AND DISCUSSION

### 3.1 Ice cream mix texture

Firmness, consistency, cohesiveness and index of viscosity values of ice cream mix samples differed significantly among all samples (*p* < .05). Interaction between samples diversity was very significant on firmness, consistency, cohesiveness, and index of viscosity values; additionally, the consistency value was negative, and the cohesiveness and index of viscosity values were positively correlated on the sample type. In addition, the firmness value on the sample type revealed a negative and highly correlated effect (Table 1).

**TABLE 1:** Physical analysis results of ice cream samples.

Samples	First drip (s.)	Meltdown (s.)	Overrun
Control	24.25 ± 1.06^ab^	103.50 ± 2.12^ab^	33.82 ± 0.48^e^
*Lb*. *casei* A. Ag.	25.25 ± 1.06^a^	107.00 ± 1.41^a^	34.47 ± 0.59^de^
*Lb*. *rhamnosus* A. Ag.	23.75 ± 1.06^abc^	102.50 ± 0.71^bc^	34.83 ± 0.26^de^
*Lb*. *lactis* A. Ag.	22.75 ± 0.35^bc^	99.00 ± 1.41^cd^	35.83 ± 0.37^cd^
*Lb*. *rhamnosus* B. Ag.	22.25 ± 0.35^cd^	98.50 ± 0.71^de^	36.93 ± 0.52^c^
*Lb*. *lactis* B. Ag.	19.75 ± 0.35^e^	93.50 ± 2.12^f^	40.88 ± 0.32^a^
*Lb*. *casei* B. Ag.	20.50 ± 0.71^de^	95.00 ± 1.41^ef^	38.76 ± 0.64^b^
*p* Value	.02	<.0001	<.0001
*r*	−.883**	−.884**	.885**

*Note*: a–f (↓): Values with the same capital letters in the same column for each analysis differ significantly (*p* < .05). *p* < .0001: Statistically too much significant, *p* < .01: Statistically too significant. **Correlation is significant at the 0.01 level (2‐tailed).

Abbreviations: A. Mat, After Maturation; B. Mat, Before Maturation.

The structural characteristics of ice cream are connected to the firmness value. After the addition of lactic acid bacteria, the results for the mixed samples' firmness and consistency fell (*p* < .05). The degree of this decrease was more significant in the samples to which lactic acid bacteria were added before mix ripening compared to the samples to which lactic acid bacteria were added after the mix matured (*p* < .05). The samples that had *L*. *lactis* introduced during either production procedure had the greatest decline. Firmness and consistency values varied between 15.11–16.26 (g) and 374.58–404.91 (g s), respectively, in the samples to which lactic acid bacteria were added before maturation; however, from the ice cream samples to which lactic acid bacteria were added after the mix matures, it varied in the ranges of 16.96–17.58 (g) and 421.48–438.89 (g s) (Table 1). Sucrose and lactose, two common sugars, have a positive impact on the mixture's ability to hold water. The environment's carbohydrates were partially digested by the lactic acid bacteria introduced to the mixture, which reduced the mixture's ability to hold water and, as a result, the hardness and consistency values. The inclusion of lactic acid bacteria reduced the cohesiveness and index of viscosity values of ice cream mixtures made in two different ways (*p* < .05). This decline was more pronounced in samples where lactic acid bacteria had been added before the mixture had fully matured (*p* < .05).

Honey and sugar increased the ice cream's stickiness and viscosity. Carbohydrates also resulted in an increasing effect (Ozdemir et al., 2008). Parallel to our research results, Alamprese et al. (2005) and Zhang et al. (2017) displayed that *L*. *plantarum* GG strains added to ice cream production changed all textural ice cream's characteristics.

The pH, a_w_, and lactic acid bacteria counts of the samples are shown in Table 1. It was determined that the interaction of sample diversity was very significant on pH and lactic acid bacteria number and water activity value. Also, it was shown that the sample interaction had very negative and highly correlative effect impact on water activity (Table 2).

**TABLE 2:** Textural analysis results of ice cream samples.

Samples	Firmness (g)	Consistency (g s)	Cohesiveness (g)	Index of viscosity (g s)
Control	17.91 ± 0.03^a^	445.44 ± 2.81^a^	−18.56 ± 0.24^d^	−29.37 ± 0.72^e^
*Lb*. *casei* A. Ag.	16.26 ± 0.21^cd^	404.91 ± 2.69^c^	−16.41 ± 0.45^b^	−20.44 ± 0.91^b^
*Lb*. *rhamnosus* A. Ag.	15.11 ± 0.40^e^	374.58 ± 7.48^d^	−14.99 ± 0.43^a^	−15.59 ± 1.91^a^
*Lb*. *lactis* A. Ag.	15.63 ± 0.66^de^	396.23 ± 13.76^c^	−15.34 ± 0.66^a^	−14.99 ± 0.98^a^
*Lb*. *rhamnosus* B. Ag.	17.58 ± 0.18^ab^	438.89 ± 7.14^a^	−17.92 ± 0.21^cd^	−27.06 ± 0.50^de^
*Lb*. *lactis* B. Ag.	16.96 ± 0.07^bc^	421.48 ± 2.51^b^	−17.29 ± 0.26^bc^	−24.43 ± 0.60^c^
*Lb*. *casei* B. Ag.	17.46 ± 0.23^ab^	433.20 ± 3.05^ab^	−17.63 ± 0.16^cd^	−26.15 ± 1.10^cd^
*p* Value	<.0001	<.0001	<.0001	<.0001
*r*	−.920*	−.898**	.940**	.958**

*Note*: **Correlation is significant at the 0.01 level (2‐tailed). *Correlation is significant at the 0.05 level (2‐tailed), a–e (↓): Values with the same capital letters in the same column for each analysis differ significantly (*p* < .05). *p* < .0001: Statistically too much significant, *p* < .01: Statistically too significant; *p* < .05: Statistically significant; *p* > .05: Not statistically significant; *p < .05; **p < .01.

Abbreviations: A. Mat, After Maturation, B. Mat, Before Maturation; ns, Not statistically significant.

### 3.2 pH and a_W_ values

The pH value within the sample reduced according to the added lactic acid bacteria species (*p* < .05). It was determined that the pH values of the samples inoculated with lactic acid bacteria before mixing maturation from two different production methods decreased more than the method in which lactic acid bacteria were added after musk maturation (*p* < .05). The samples with lactic acid bacteria introduced before mix maturation had pH values ranging from 6.39 to 6.46, while those with lactic acid bacteria added after mix maturation had pH values ranging from 6.51 to 6.54. The samples created by adding three distinct lactic acid bacteria had pH values that were lower than the control sample. This difference is caused by the organic acids formed due to the metabolization of the hexose sugars in the mix by the added lactic acid bacteria. It was determined that the pH values of the samples inoculated with lactic acid bacteria before the ripening of the mix from two different production methods decreased more compared to the method in which lactic acid bacteria was added after the musk ripening. Similar to our research results, Zhang et al. (2014), Sarwar et al. (2021), and Goktas et al. (2022) declared that the pH levels of their samples were lowered by the addition of probiotic bacteria. According to the added lactic acid bacteria species in the current investigation, the water activity values of the samples altered (*p* < .05). The water activity value in the samples to which lactic acid bacteria were added before musk maturation was less than those in which lactic acid bacteria were added after mix maturation (*p* < .05). Although the a_w_ values in the specimens after maturation varied between 0.726 and 0.730 (*p* > .05), it showed a change in the range of 0.717–0.722 in the samples to which bacteria were added before maturation (*p* < .05). The lactic acid bacteria numbers in the samples varied according to the added lactic acid bacteria (*p* < .05). In comparison to samples added after mix maturation, the samples to which lactic acid bacteria were introduced had a greater lactic acid bacteria count (*p* < .05). It was determined that samples containing *L. lactis* had the highest bacterial count in both production methods.

### 3.3 Lactic acid bacteria count

The optimum temperatures in order for lactic acid bacteria to develop belonging to the genus *Lactobacillus* are between 30 and 40°C. Its growth temperature range is 2–53°C (Idler et al., 2015). In addition, the medium's pH, a_w_, O/R potential, and viscosity. The bacteria can also develop at lower temperatures if the conditions are suitable (Novak et al., 1997). The generation time increases as the ambient temperature moves away from the optimum temperature (Adams & Moss, 2015). At lower temperatures, the bacteria can survive even if they cannot thrive (Idler et al., 2015). According to studies, it has been observed that *L*. *lactis* can grow at lower temperatures than other lactic acid bacteria (Novak et al., 1997). The findings presented by the researchers agree with our research results. In ice cream production, lactic acid bacteria were inoculated into the mixes at an average of 10^8^–10^9^ log cfu/g.

Before maturation, the mixture was cooled to 37°C and infected with lactic acid bacteria. The mixtures were then allowed to mature at 4°C for 24 h. The number of lactic acid bacteria increased until the mix temperature dropped below the minimum limit at which lactic acid bacteria could grow. In the other production method, bacteria were added to the mix after the maturation process, and the mixture was then converted into ice cream. Since the time elapsed was not very long, we did not find enough time to increase the number of bacteria in this type of production. In both production methods, some of the bacteria were not affected by the very low temperature during the ice cream production stage and could not survive.

### 3.4 Color (L*, a*, and b*) values

One of the most crucial food quality parameters in the eyes of the consumer is color values (L*: Brightness, a*: Redness, and b*: Yellowness) (Chranioti et al., 2015). The a* value of the ice cream sample is positively and significantly correlated with the sample type.

**FIGURE 2:**
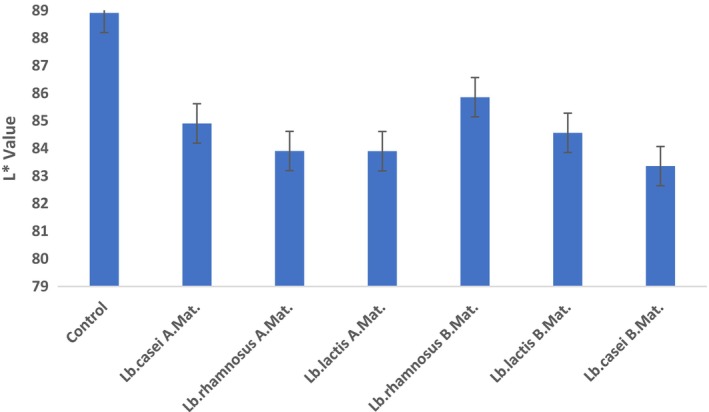
L* values of the samples. A. Mat, After Maturation; B. Mat, Before Maturation.

**FIGURE 3:**
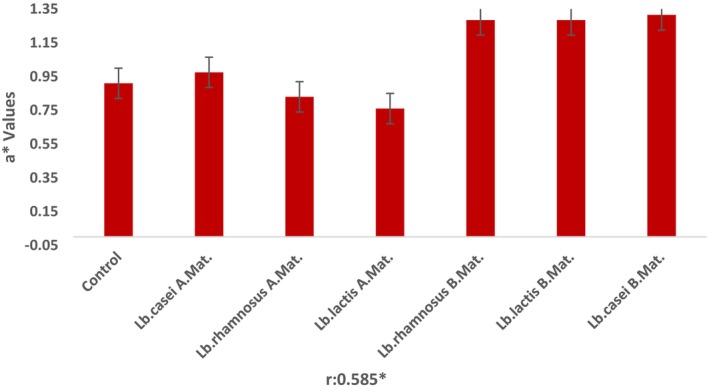
a* Values of the samples. A. Mat, After Maturation; B. Mat, Before Maturation. *Correlation is significant at the 0.05 level (2‐tailed).

**FIGURE 4:**
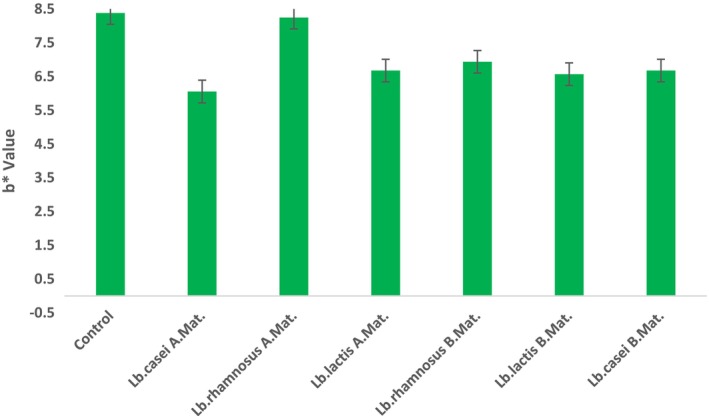
b* Values of the Samples. A. Mat, After Maturation; B. Mat, Before Maturation.

No significant effect of the addition of lactic acid bacteria to the mix or the addition of bacteria before or after the maturation of the mixture on the L*, a*, and b* values was detected (*p* > .05). Although the a* values of the samples to which lactic acid bacteria were added after maturation were found to be higher than the samples added before maturation, this distinction is not statistically noteworthy (*p* > .05). The L* values of the samples varied between 88.91 and 83.36 (Figure 2), a* values between 0.76 and 1.32 (Figure 3), and b* values between 6.57 and 8.38 (Figure 4). In parallel with our findings, Kilinc and Sevik (2021) found that the L* value of samples was found to be 91.11, means a*; 1.39 and b*. The authors stated that they determined it as 8.07.

### 3.5 Overrun, first dripping, and complete melting time

The relationship between the sample types was extremely important on the total melting and overrun values, as well as on the first drop, according to the findings of the variation analysis performed on the physical values of the samples. Moreover, it was revealed that the sample type had a negative effect on the first drip and complete melting and a positive and very highly correlative effect on the overrun (Table 3). Ice cream's melting behavior is described as an empirical attribute that reflects how resistant it is to melt in warm environments and is observed to be highly correlated with the material's thermal conductivity, heat capacity, and microstructure (Sun‐Waterhouse et al., 2013). The first dripping and full melting times of the ice cream were shortened by adding lactic acid bacteria to the mixture (*p* < .05). This decrease was more pronounced in the samples to which lactic acid bacteria were added before the mix was matured compared to the samples added after the mix was matured (*p* < .05). In both production processes for the two analyses, the samples with additional *L*. *lactis* showed the greatest decrease. Each starter culture may have different effects on the melting characteristics of ice cream and the degree of this effect may differ. However, starter and non‐starter cultures cannot affect ice cream's melting properties (Alamprese et al., 2005). The amount of air in the mixture, the makeup of the mixture, the network of fat globules, and the ice crystal structure generated during ice cream production are just a few of the many variables that affect how quickly ice cream melts. Due to their ability to store more water and their ability to create micro‐viscosity, sugars and lactose added to the mixture increase the melting resistance of ice cream (Bahram Parvar & Tehrani, 2011; Muse & Hartel, 2004). In this investigation, lactic acid bacteria were added to the ice cream mixture, and they digested the carbs, breaking down the milk proteins and releasing lactic acid. Organic acid production increased, which led to a decline in melting resistance. Since adding bacteria before mix maturation increased the amount of metabolized sugar, dissolution resistance decreased in these samples.

**TABLE 3:** Physicochemical and microbiological properties of ice cream samples.

Samples	pH	a_w_	Bacteria count (log cfu/g)
Control	6.56 ± 0.01^a^	0.729 ± 0.001^a^	0.00 ± 0.00^f^
*Lb*. *casei* A. Ag.	6.44 ± 0.01^d^	0.726 ± 0.001^a^	10.13 ± 0.03^b^
*Lb*. *rhamnosus* A. Ag.	6.46 ± 0.01^d^	0.728 ± 0.001^a^	9.82 ± 0.04^c^
*Lb*. *lactis* A. Ag.	6.39 ± 0.01^e^	0.730 ± 0.001^a^	11.35 ± 0.04^a^
*Lb*. *rhamnosus* B. Ag.	6.54 ± 0.01^ab^	0.717 ± 0.003^c^	8.44 ± 0.08^d^
*Lb*. *lactis* B. Ag.	6.51 ± 0.01^b^	0.719 ± 0.002^bc^	8.89 ± 0.02^c^
*Lb*. *casei* B. Ag.	6.52 ± 0.00^bc^	0.722 ± 0.001^b^	7.63 ± 0.02^d^
*p* Value	<.0001	.01	<.0001
*r*	.189	−.686**	.004

*Note*: a–f (↓): Values with the same capital letters in the same column for each analysis differ significantly (*p* < .05). **Correlation is significant at the 0.01 level (2‐tailed), *p* < .0001: Statistically too much significant; *p* < .01: Statistically too significant; *p* < .05: Statistically significant; *p* > .05: Not statistically significant.

Abbreviations: A. Ag, After Aging; B. Ag, Before Aging; ns, Not statistically significant.

Overrun is the quantity of air added to the mixture during the process of turning it into ice cream (Cruz et al., 2009). In the current investigation, lactic acid bacteria were added to the ice cream mixtures to reduce overflow (*p* < .05). This decrease occurred more in the samples to which bacteria were added before maturation than those added after maturation (*p* < .05). It was shown that among the samples, those made with the inclusion of *L*. *lactis* had the greatest drop in overrun value.

In previous studies, Abghari et al. (2011), Sarwar et al. (2021), and Goktas et al. (2022) reported that they found similar results. In our study, the lactic acid bacteria added to the mixes metabolized the sugars in the medium, causing a decrease in the mix viscosity and, accordingly, a decrease in the amount of overrun. There are also negative effects of the decrease in pH on overrun (Allen et al., 2006).

### 3.6 Organic acid amount of samples

The organic acid amounts of the samples are shown in Table 4. It was determined that the interaction of sample diversity was very significant on lactic, citric, succinic, malic, oxalic, tartaric, and formic acids, which were organic acids in the ice cream samples and very significant on fumaric acid. Moreover, sample diversity had a favorable and highly correlated influence on succinic acid and a detrimental and correlated effect on citric and formic acids (Table 4). The addition of lactic acid bacteria enhanced the samples' levels of organic acids (apart from formic acid) (*p* < .05). This rise was much higher in the samples to which lactic acid bacteria were added before mix maturation compared to the samples to which lactic acid bacteria were added after maturation (*p* < .05). The organic acids with the highest increase were determined as tartaric, succinic, and lactic acids, while the highest gains were determined in the samples created after *L*. *lactis* was added. In addition, although malic acid was detected only in the samples to which lactic acid bacteria were added before ripening, formic acid was detected only in the control sample. The main products of lactic acid bacteria's metabolism of carbohydrates are organic acids. They occur when glucose in the environment is metabolized by lactic acid bacteria in homo‐ and heterofermentative ways (Sun et al., 2014).

**TABLE 4:** Organic acid content of ice cream samples (mg/kg).

Samples	Ascorbic acid	Lactic acid	Citric acid	Succinic acid	Malic acid
Control	30.45 ± 0.17^ab^	942.68 ± 6.02^e^	127.83 ± 6.34^a^	1885.70 ± 74.88^e^	0.00 ± 0.00^c^
*Lb*. *casei* A. Ag.	33.05 ± 8.76^a^	564.40 ± 14.06^f^	83.371 ± 5.73^b^	3357.23 ± 21.35^cd^	8.47 ± 0.17^b^
*Lb*. *rhamnosus* A. Ag.	27.92 ± 5.11^abc^	3950.78 ± 71.48^b^	84.827 ± 6.50^b^	2273.74 ± 17.68^e^	8.28 ± 0.18^b^
*Lb*. *lactis* A. Ag.	21.17 ± 0.57^bc^	4610.02 ± 63.36^a^	88.018 ± 4.25^b^	2843.28 ± 84.80^d^	10.42 ± 0.04^a^
*Lb*. *rhamnosus* B. Ag.	20.25 ± 0.33^bc^	1138.35 ± 43.81^d^	41.646 ± 5.10^e^	3627.29 ± 371.86^c^	0.00 ± 0.00^c^
*Lb*. *lactis* B. Ag.	29.87 ± 5.02^abc^	1328.28 ± 38.13^c^	34.897 ± 4.15^e^	6150.31 ± 205.70^b^	0.00 ± 0.00^c^
*Lb*. *casei* B. Ag.	18.83 ± 3.84^c^	1259.29 ± 21.13^c^	67.393 ± 7.85^c^	12737.32 ± 388.90^a^	0.00 ± 00^c^
*p* Value	.78	<.0001	<.0001	<.0001	<.0001
*r*	−.388	−.016	−.783*	.800**	−.398

Samples	Oxalic acid	Fumaric acid	Tartaric acid	Formic acid
Control	0.53 ± 0.04^g^	2.06 ± 0.04^d^	175.09 ± 6.44^g^	455.995 ± 6.30^a^
*Lb*. *casei* A. Ag.	3.04 ± 0.10^b^	2.48 ± 0.14^a^	16589.74 ± 20.36^a^	0.00 ± 0.00^b^
*Lb*. *rhamnosus* A. Ag.	1.18 ± 0.06^e^	2.27 ± 0.06^bc^	2497.21 ± 20.41^c^	0.00 ± 0.00^b^
*Lb*. *lactis* A. Ag.	8.33 ± 0.03^a^	2.53 ± 0.02^a^	1823.76 ± 19.96^e^	0.00 ± 0.00^b^
*Lb*. *rhamnosus* B. Ag.	0.76 ± 0.02^f^	2.11 ± 0.07^cd^	2066.37 ± 18.94^d^	0.00 ± 0.00^b^
*Lb*. *lactis* B. Ag.	1.51 ± 0.02^c^	2.39 ± 0.08^ab^	758.56 ± 7.88^f^	0.00 ± 0.00^b^
*Lb*. *casei* B. Ag.	1.33 ± 0.01^d^	2.12 ± 0.04^cd^	4394.27 ± 121.08^b^	0.00 ± 0.00^b^
*p* Value	<.0001	.02	<.0001	<.0001
*r*	−.031	−.063	−.263	−.612*

*Note*: a–f (↓): Values with the same capital letters in the same column for each analysis differ significantly (*p* < .05). **Correlation is significant at the 0.01 level (2‐tailed). *Correlation is significant at the 0.05 level (2‐tailed), *p* < .0001: Statistically too much significant; *p* < .01: Statistically too significant; *p* < .05: Statistically significant; *p* > 0.05: Not statistically significant; *p < .05; **p < .01.

Abbreviations: A. Ag, After Aging, B. Ag, Before Aging; ns, Not statistically significant.

### 3.7 Fatty acid distributions of ice cream samples

Table 5 displays the ice cream sample fatty acid distributions. The conclusion was made that the interaction of sample type was very significant on the amounts of all other fatty acids, except for capric, tridecanoic, eicosanoic, docosanoic, and linolenic acids. In addition, the sample type lauric, palmitic, oleic, and linoleic acids were positively correlated—excessively with respect to palmitoleic and γ‐linolenic acids and negatively correlated with capric acid (Table 5). The amounts of fatty acids varied depending on the addition of lactic acid and the production method (*p* < .05). Amount of this change was generally higher in the samples to which lactic acid bacteria were added after mix maturation. When the distribution of detected fatty acids was examined, it was revealed that the amount of saturated fatty acid was higher than the amount of unsaturated fatty acid and the amount of medium‐chain fatty acid was higher than the amount of short and long‐chain fatty acids in all samples. The highest amount of saturated fatty acid in both production forms was 66.308% and 66.984%, respectively, the samples created using the addition of *L*. *rhamnosus*. The highest amount of saturated fatty acids were detected in samples produced with the addition of *L. rhamnosus*, with 66.308% and 66.984%, respectively, in both production methods. The least saturated fatty acid is; Again, in both production methods, it was detected in samples produced with the addition of L. casei at 63.623% and 66.167%, respectively. The highest medium chain fatty acid was determined at 48.882% in the product produced with the addition of *L*. *lactis* after mix ripening.

**TABLE 5:** Distribution of % fatty acids of ice cream samples.

	Saturated fatty acids				
	Butanoic acid (C4.0)	Caproic acid (C6.0)	Caprylic acid (C8.0)	Capric acid (C10.0)	Lauric acid (C12.0)
RT (s.)	6.211	10.517	18.017	28.085	38.832
Control	0.518 ± 0.006^c^	0.886 ± 0.005^bc^	0.728 ± 0.007^ab^	1.77 ± 0.006 cd	2.329 ± 0.04^d^
*Lb*. *casei* A. Mat.	0.405 ± 0.006^d^	0.721 ± 0.007^f^	0.603 ± 0.007^d^	1.57 ± 0.007e	2.495 ± 0.03^b^
*Lb*. *rhamnosus* A. Mat.	0.568 ± 0.004^b^	0.867 ± 0.011^c^	0.709 ± 0.016^bc^	1.80 ± 0.028bc	2.463 ± 0.02^b^
*Lb*. *lactis* A. Mat.	0.334 ± 0.003^e^	0.847 ± 0.007^d^	0.708 ± 0.011^bc^	1.75 ± 0.008d	2.394 ± 0.02^c^
*Lb*. *rhamnosus* B. Mat.	0.582 ± 0.003^a^	0.896 ± 0.006^b^	0.725 ± 0.007^ab^	1.84 ± 0.008a	2.486 ± 0.02^b^
*Lb*. *lactis* B. Mat.	0.558 ± 0.007^b^	0.952 ± 0.011^a^	0.753 ± 0.018^a^	1.81 ± 0.012ab	2.469 ± 0.02^b^
*Lb*. *casei* B. Mat.	0.409 ± 0.007^d^	0.813 ± 0.007^e^	0.693 ± 0.010^c^	1.82 ± 0.011ab	2.647 ± 0.02^a^
*p* Value	<.0001	<.0001	<.0001	.038	<.0001
*r*	−.006	−.198	.334	−.623*	.717**

	Saturated fatty acids				
	Tridecanoic acid (C13.0)	Myristic acid (C14.0)	Pentadecanoic acid (C15.0)	Pentadecanoic acid. 14‐methyl	Palmitic acid (C16.0)
RT (s.)	44.044	46.749	51.735	56.531	59.031
Control	0.076 ± 0.007^a^	10.107 ± 0.05^f^	1489 ± 0.009^a^	0.374 ± 0.005^a^	35.115 ± 0.03^e^
*Lb*. *casei* A. Mat.	0.063 ± 0.005^a^	10.327 ± 0.01^c^	1287 ± 0.004^d^	0.302 ± 0.008^d^	35.134 ± 0.06^e^
*Lb*. *rhamnosus* A. Mat.	0.068 ± 0.003^a^	10.444 ± 0.02^b^	1470 ± 0.012^ab^	0.352 ± 0.006^b^	35.331 ± 0.06^d^
*Lb*. *lactis* A. Mat.	0.068 ± 0.008^a^	10.026 ± 0.02^e^	1409 ± 0.013^c^	0.332 ± 0.007^c^	35.186 ± 0.10^e^
*Lb*. *rhamnosus* B. Mat.	0.071 ± 0.008^a^	10.488 ± 0.01^b^	1472 ± 0.008^ab^	0.352 ± 0.003^b^	35.693 ± 0.02^c^
*Lb*. *lactis* B. Mat.	0.069 ± 0.006^a^	10.159 ± 0.01^d^	1466 ± 0.005^ab^	0.343 ± 0.010^bc^	36.145 ± 0.04^a^
*Lb*. *casei* B. Mat.	0.070 ± 0.007^a^	10.498 ± 0.01^a^	1415 ± 0.009^b^	0.344 ± 0.006^bc^	35.798 ± 0.03^b^
*p* Value	.676	<.0001	<.0001	<.0001	<.0001
*r*	−.036	.517	.295	−.027	.819**

	Saturated fatty acids				Monounsaturated fatty acids
	Heptadecanoic acid (C17.0)	Stearic acid (C18.0)	Eicosanoic acid (C20.0)	Docosanoic acid (C22.0)	Palmitoleic acid (C16.1)
RT (s.)	59.905	67.858	75.882	83.54	59.904
Control	0.767 ± 0.007^a^	11.304 ± 0.007^a^	0.207 ± 0.010^a^	0.096 ± 0.003^ab^	2476 ± 0.006^cd^
*Lb*. *casei* A. Mat.	0.644 ± 0.003^d^	9816 ± 0.014^f^	0.180 ± 0.028^a^	0.076 ± 0.006^c^	2484 ± 0.006^c^
*Lb*. *rhamnosus* A. Mat.	0.746 ± 0.004^b^	11.197 ± 0.015^b^	0.199 ± 0.007^a^	0.094 ± 0.011^ab^	2480 ± 0.007^c^
*Lb*. *lactis* A. Mat.	0.729 ± 0.006^c^	10.537 ± 0.011^e^	0.181 ± 0.006^a^	0.074 ± 0.006^c^	2449 ± 0.011^e^
*Lb*. *rhamnosus* B. Mat.	0.753 ± 0.004^b^	11.301 ± 0.020^a^	0.217 ± 0.004^a^	0.108 ± 0.004^a^	2493 ± 0.004^b^
*Lb*. *lactis* B. Mat.	0.730 ± 0.006^c^	10.761 ± 0.005^c^	0.187 ± 0.004^a^	0.082 ± 0.004^bc^	2455 ± 0.008^de^
*Lb*. *casei* B. Mat.	0.721 ± 0.007^c^	10.677 ± 0.011^d^	0.184 ± 0.065^a^	0.078 ± 0.003^c^	2647 ± 0.016^a^
*p* Value	<.0001	<.0001	.759	.05	<.0001
*r*	.079	.089	−.113	−.160	.614*

RT (s.)	Monounsaturated fatty acids	Polyunsaturated fatty acids			
	Oleic acid (C18.1)	Myristoleic acid (C14.2)	Linoleic acid (C18.2)	γ‐Linolenic acid (C18.3)	Linolenic acid (C18.3)
Control	68.61	50.878	70.344	71.845	73.985
*Lb*. *casei* A. Mat.	24.854 ± 0.008^e^	1982 ± 0.009^a^	2152 ± 0.006^d^	0.433 ± 0.006^e^	0.581 ± 0.007^a^
*Lb*. *rhamnosus* A. Mat.	25.227 ± 0.014^c^	1803 ± 0.014^c^	2105 ± 0.011^e^	0.510 ± 0.019^c^	0.547 ± 0.006^b^
*Lb*. *lactis* A. Mat.	24.888 ± 0.017^e^	1845 ± 0.017^b^	2056 ± 0.011^f^	0.450 ± 0.005^d^	0.591 ± 0.008^a^
*Lb*. *rhamnosus* B. Mat.	25.018 ± 0.013^d^	1288 ± 0.011^d^	1979 ± 0.011^g^	0.424 ± 0.007^e^	0.583 ± 0.010^a^
*Lb*. *lactis* B. Mat.	25.026 ± 0.007^d^	1852 ± 0.014^b^	2357 ± 0.013^b^	0.555 ± 0.010^b^	0.592 ± 0.004^a^
*Lb*. *casei* B. Mat.	26.02 ± 0.008^a^	1795 ± 0.013^c^	2177 ± 0.016^c^	0.452 ± 0.003^d^	0.591 ± 0.007^a^
*p* Value	25.335 ± 0.32^b^	1865 ± 0.024^b^	2544 ± 0.024^a^	0.852 ± 0.004^a^	0.587 ± 0.010^a^
*r*	<.0001	<.0001	<.0001	<.0001	.006
	.691**	−.120	.669**	.635*	.491

Samples	SFA	USFA	SCFA	MCFA	LCFA
Control	65.766	32.478	6.23	47.161	39.627
*Lb*. *casei* A. Mat.	63.623	32.676	5.79	47.113	38.461
*Lb*. *rhamnosus* A. Mat.	66.308	32.31	6.41	47.664	39.475
*Lb*. *lactis* A. Mat.	64.575	31.741	6.03	47.021	38.796
*Lb*. *rhamnosus* B. Mat.	66.984	32.875	6.53	48.076	40.361
*Lb*. *lactis* B. Mat.	66.484	33.49	6.54	48.882	41.022
*Lb*. *casei* B. Mat.	66.167	33.83	6.38	48.576	42.357

*Note*: a–f (↓): Values with the same capital letters in the same column for each analysis differ significantly (*p* < .05). **Correlation is significant at the 0.01 level (2‐tailed). *Correlation is significant at the 0.05 level (2‐tailed), *p* < .0001: Statistically too much significant, *p* < .01: Statistically too significant; *p* < .05: Statistically significant; *p* > .05: Not statistically significant; *p < .05; **p < 0.01.

Abbreviations: A. Mat, After Maturation; B. Mat, Before Maturation; A. Ag, After Aging; B. Ag, Before Aging; CLA, Conjugated linolenic acid; LCFA, Long chain fatty acids (≥C:18); MCFA, Medium chain fatty acids (C:14–C:16); ns, Not statistically significant; RT, Retention Time; SCFA, Short chain fatty acids (C:4–C:12); SFA, Saturated fatty acids; USFA, Unsaturated fatty acids.

## 4 CONCLUSION

This study investigated ice cream's physicochemical and technological properties by adding different lactic acid bacteria using two different production methods. Adding lactic acid bacteria to the mix caused a decrease in firmness, consistency, cohesiveness, index of viscosity, pH, a_w_ first drop, complete melting, and overrun values. These decreases were especially higher in the samples to which lactic acid bacteria were added before mix maturation. In addition, it was found that samples created by incorporating lactic acid bacteria had more organic acid (apart from formic acid). Also, the addition of lactic acid and the technique of manufacture affected the amount of fatty acids in the ice cream samples; the rate of change was often larger in the samples that included lactic acid bacteria following mix ripening. Particularly, in the samples with lactic acid bacteria introduced after mix maturation as compared to the control sample, the levels of short‐ and medium‐chain fatty acids increased. Consumer demand for foods with better functional qualities is increasing today. The results of this study suggest that, because of fermenting the ice cream mix with lactic acid bacteria, it becomes a product richer in terms of physicochemical properties and nutritional values. It has been demonstrated that incorporating lactic acid bacteria into two distinct industrial processes, especially after mix maturation, is more effective on the results.

The findings from this study reveal that many different combinations of bacteria and milk varieties can be used in the production of ice cream in the future. By this means, it is obvious that it will enable to obtain more functional, nutritionally rich, and useful products. In addition, it is thought that a new page will be opened in the ice cream industry with the production of these products.

## AUTHOR CONTRIBUTIONS

**Gokhan Akarca:** Conceptualization (lead); data curation (equal); investigation (equal); methodology (lead); project administration (lead); resources (equal); writing – original draft (lead); writing – review and editing (equal). **Mehmet Kılınç:** Formal analysis (equal); investigation (equal); resources (equal); writing – review and editing (equal). **Ayşe Janseli Denizkara:** Data curation (equal); formal analysis (equal); resources (equal); writing – review and editing (equal).

## FUNDING INFORMATION

The authors declare that no funds, grants, or other support were received during the preparation of this manuscript.

## CONFLICT OF INTEREST STATEMENT

The authors declared no potential conflicts of interest with respect to the research, authorship, and/or publication of this manuscript.

## ETHICS STATEMENT

This article does not contain any studies with human participants or animals performed by any of the authors.

## CONSENT TO PARTICIPATE

The corresponding author and all the co‐authors participated in the preparation of this manuscript.

## INFORMED CONSENT

For this type of study, formal consent is not required.

## Data Availability

The original data with the respective analysis corresponding to the results shown in this work are available up to reasonable requirements.
